# A label‐free impedance assay in endothelial cells differentiates the activation and desensitization properties of clinical S1P_1_ agonists

**DOI:** 10.1002/2211-5463.12951

**Published:** 2020-09-04

**Authors:** Patrick Grailhe, Asma Boutarfa‐Madec, Philippe Beauverger, Philip Janiak, Ashfaq A. Parkar

**Affiliations:** ^1^ Diabetes and Cardiovascular Research Sanofi R&D Chilly‐Mazarin France; ^2^ Diabetes and Cardiovascular Research Sanofi US Services Bridgewater NJ USA

**Keywords:** desensitization, endothelium, impedance, S1P_1_, SAR247799, siponimod

## Abstract

Sphingosine‐1 phosphate receptor‐1 (S1P_1_) activation maintains endothelial barrier integrity, whereas S1P_1_ desensitization induces peripheral blood lymphopenia. The latter is exploited in the approval and/or late‐stage development of receptor‐desensitizing agents targeting the S1P_1_ receptor in multiple sclerosis, such as siponimod, ozanimod, and ponesimod. SAR247799 is a recently described G protein‐biased S1P_1_ agonist that activates S1P_1_ without desensitization and thus has endothelial‐protective properties in patients without reducing lymphocytes. As SAR247799 demonstrated endothelial‐protective effects at sub‐lymphocyte‐reducing doses, the possibility exists that other S1P_1_ modulators could also exhibit endothelial‐protective properties at lower doses. To explore this possibility, we sought to quantitatively compare the biased properties of SAR247799 with the most advanced clinical molecules targeting S1P_1_. In this study, we define the β‐arrestin pathway component of the impedance profile following S1P_1_ activation in a human umbilical vein endothelial cell line (HUVEC) and report quantitative indices of the S1P_1_ activation‐to‐desensitization ratio of various clinical molecules. In a label‐free impedance assay assessing endothelial barrier integrity and disruption, the mean estimates (95% confidence interval) of the activation‐to‐desensitization ratios of SAR247799, ponesimod, ozanimod, and siponimod were 114 (91.1–143), 7.66 (3.41–17.2), 6.35 (3.21–12.5), and 0.170 (0.0523–0.555), respectively. Thus, we show that SAR247799 is the most G protein‐biased S1P_1_ agonist currently characterized. This rank order of bias among the most clinically advanced S1P_1_ modulators provides a new perspective on the relative potential of these clinical molecules for improving endothelial function in patients in relation to their lymphocyte‐reducing (desensitization) properties.

## Graphical abstract

S1P_1_ activation maintains endothelial barrier integrity, whereas its desensitization induces lymphopenia. SAR247799, a new G protein‐biased S1P_1_ agonist, was compared to clinical stage S1P_1_‐desensitizing compounds using a label‐free impedance assay assessing endothelial barrier integrity. SAR247799 had the highest activation‐to‐desensitization ratio (114), compared to ponesimod (7.66), ozanimod (6.35), and siponimod (0.170) and thus demonstrated the best ability to cause sustained S1P_1_ activation.

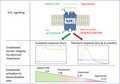

**Abbreviations**
AUC: area under curve
BBB: blood–brain barrier
BNCI: baseline normalized cell index
cAMP: cyclic adenosine monophosphate
CNS: central nervous system
FBS: fetal bovine serum
GPCR: G protein‐coupled receptor
GRK: G protein‐coupled regulated kinase
HUVEC: human umbilical vein endothelial cell
MS: multiple sclerosis
RTCA: real‐time cellular assay
S1P: sphingosine‐1 phosphate
S1P_1_: S1P receptor‐1

Sphingosine‐1 phosphate receptor‐1 (S1P_1_) is a G protein‐coupled receptor of the sphingolipid family [1]. S1P_1_ activation causes GTP/GDP exchange in a Gαi‐dependent manner, resulting in the inhibition of cyclic adenosine monophosphate (cAMP) generation [2]. S1P_1_ can also signal through recruitment of β‐arrestin causing receptor internalization and subsequent desensitization of G protein‐mediated responses. Compounds targeting this receptor have been primarily developed as receptor‐desensitizing agents with associated peripheral blood lymphopenia, and this has been exploited in the approval of 3 drugs for multiple sclerosis, fingolimod (a nonselective S1P_1/3/4/5_ agonist), and more recently siponimod and ozanimod (S1P_1/5_ agonists) [3, 4, 5]. S1P_1_‐desensitizing molecules in clinical development include ponesimod (in phase 3 trials for MS) as well as molecules that have shown efficacy in other autoimmune diseases including inflammatory bowel disease, lupus, and psoriasis [6, 7, 8]. S1P_1_ activation has endothelial barrier‐stabilizing effects through the formation of adherens and tight junctions [9, 10]. Recently, we reported the discovery of SAR247799 a G protein‐biased S1P_1_ selective agonist capable of S1P_1_ activation while limiting receptor desensitization [11]. The biased properties of SAR247799 were associated, in rat and pig models of ischemia/reperfusion injury, with endothelial‐protective properties at doses that did not show lymphocyte reduction, and lymphopenia was only evident at supratherapeutic doses [11]. Similarly, 5‐week sustained activation of S1P_1_ in diabetic rats showed improvements in renal function and endothelial function without causing receptor desensitization [12]. Furthermore, these preclinical findings showed translation to human studies, where SAR247799 showed improvement in endothelial function in type‐2 diabetes patients, again at sub‐lymphocyte‐reducing doses [12]. SAR247799 displayed an attractive safety and tolerability profile in humans, and supratherapeutic doses were characterized by dose‐dependent lymphocyte reduction, a biphasic effect consistent with that observed in preclinical studies [11, 12, 13].

As SAR247799 demonstrated endothelial‐protective effects at sub‐lymphocyte‐reducing doses, the possibility exists that other S1P_1_ modulators, although developed as S1P_1_‐desensitizing molecules, might also exhibit endothelial‐protective properties at lower doses. To explore this possibility, we sought to quantitatively compare the biased properties of SAR247799 with the most advanced clinical molecules targeting S1P_1_ in a relevant endothelial cell‐based assay.

We previously reported the biased properties of SAR247799 by measuring the potency and efficacy for activation of G protein pathways (inhibition of forskolin‐induced cAMP) relative to β‐arrestin recruitment and receptor internalization pathways [11]. SAR247799 displays more G protein‐biased S1P_1_ agonist properties than siponimod in these receptor overexpression assays. However, it is important to recognize the limitations of various cell‐based assays for determining ligand bias [14]. Cell‐based assays relying on receptor overexpression may not fully recapitulate the same consequences associated with endogenous receptor signaling. The stoichiometry between receptor occupancy and intracellular events is often altered in assays that rely on biosensors, due to signal amplification. Gαi‐coupled receptors are particularly challenging because intracellular G protein signaling is measured indirectly by inhibition of forskolin‐induced cAMP production, usually in transfected cells. Furthermore, the measurement of bias requires comparison of two separate assays (e.g., cAMP with β‐arrestin recruitment or receptor internalization), and differences in timepoints and assay conditions may cause the physiochemical properties of test compounds to influence experimental readouts to different extents. Consequently, such systems can lead to under‐ or over‐reporting of receptor bias, and a previous study using various S1P_1_‐overexpressing cell assays did not find differences in signaling between molecules [15]. The ideal approach to quantify GPCR bias would be by utilizing a single assay capable of measuring activation and desensitization in the same setting, be performed in relevant cells without receptor overexpression, and not rely on reporter systems that introduce the possibility of signal amplification.

Endothelial barrier function can be measured by the passage of molecules across a cell layer [16, 17]. Movement of ions across the endothelial layer occurs mainly by intercellular exchange, and the integrity of cell‐cell junctions is the primary resistance to this movement [18]. Trans‐endothelial electrical resistance, or impedance, is therefore an index of endothelial barrier integrity. A real‐time cellular assay (RTCA) in human umbilical vein endothelial cells (HUVEC), utilizing a label‐free electrical impedance measurement, has been shown to produce a Gi‐mediated increase in impedance following activation with S1P_1_ agonists [19]. We previously reported qualitative differences in the desensitization properties of SAR247799 and siponimod using such a system [11]. We now extend these observations to define the β‐arrestin pathway component of the impedance profile following S1P_1_ activation in HUVECs, and we report quantitative indices of the S1P_1_ activation‐to‐desensitization ratio of various clinical molecules. We show that SAR247799 is the most G protein‐biased S1P_1_ agonist currently characterized and provide a rank order of bias among the most clinically advanced S1P_1_ modulators.

## Materials and methods

### Compounds

SAR247799 (4‐[5‐(3‐chloro‐phenoxy)‐oxazolo [5,4‐d]pyrimidin‐2‐yl]‐2,6‐dimethyl‐phenoxy}‐acetic acid), siponimod, and the GRK inhibitor (1H‐Indazole‐5‐carboxylic acid [3‐(2‐trifluoromethyl‐benzylamino)‐1,2‐benzisoxazol‐5‐ylmethyl]‐amide) were synthesized at Sanofi. Ponesimod, ozanimod, and S1P were purchased from Selleck Chemicals LLC (Houston, TX, USA), Apexbio Technology LLC (Houston, TX, USA), and Avanti Polar Lipids (Alabaster, AL, USA), respectively.

### Impedance protocol

HUVECs from pooled donors (PromoCell GmbH, Heidelberg, Germany, C‐12203) were seeded at 10 000 cells per well, in complete medium (C2210, C39210) containing 2% fetal bovine serum (FBS), in 96‐well collagen‐I coated E‐plates. Cells were allowed to attach and proliferate for 6 h, followed by overnight serum starvation in medium containing 0.1% FBS. Electrical impedance was measured continuously with RTCA‐MP station (xCELLigence RTCA, ACEA‐Biosciences, San Diego, CA, USA), according to the manufacturer’s protocol, and expressed as baseline normalized cell index (BNCI) using rtca2.0 software. Impedance measurements were analyzed for 60 min following addition of test compounds or DMSO control, and the early (peak response at 8–10 min) and late response (at 60 min) was used for further analysis. Cells were then washed in medium containing 0.1% FBS for 5.5 h. The effect of each test compound to desensitize the response to a second stimulation with the natural ligand S1P (80 nm) was measured in the same wells and expressed as the AUC_0–60 min_ of the S1P‐induced BNCI response. S1P (Avanti Polar Lipids) was prepared from a 125 µm stock solution in 4 mg·mL^−1^ BSA according to the manufacturer’s instructions. The baseline for the BNCI calculation was the respective vehicle responses for the first (0.1% DMSO) and second stimulations (2.5 µg·mL^−1^ BSA). When tested, 10 µm GRK2 inhibitor was pretreated with cells for 2 h prior to addition of test compounds and its effect on early and late responses measured as above. All experiments were repeated on at least 3 separate occasions.

### GRK assays

The biochemical potency on GRK2 was determined using recombinant human GRK2 (Catalogue number PR4694A, Thermo Fisher, Les Ulis, France) in a ^33^P‐ATP flash plate kinase assay with 3 µm ATP and biotin‐RRREEEEESAAA as substrate. The cellular potency of the GRK2 inhibitor was determined using a β‐arrestin recruitment assay in PathHunter^®^ cells overexpressing human S1P_1_ (Eurofins DiscoverX Corporation, San Diego, CA, USA; catalogue number 93‐0207C2) as described [11]. The mean IC_50_ from at least 3 separate experiments was reported.

### Calculations

Effective concentration corresponding to half of the difference between the maximum and minimum effect (EC_50_) of agonists was determined with SAS procedure NLIN in SAS system release 9.1 under unix via biostat@t‐speed‐lts v2.0 internal software using the 4‐parameter logistical model.

The potency of test compounds to desensitize the S1P‐induced BNCI response was determined as the inhibitory concentration corresponding to 50% of the S1P response in the absence of test compound (IC_50_) and determined using the 4‐parameter logistical model as above.

The activation‐to‐desensitization ratio was expressed as IC_50_/EC_50_ for early phase. EC_50_, IC_50_, and activation‐to‐desensitization ratio were reported as geometric mean with 95% confidence intervals.

## Results

### SAR247799 produced a sustained cell impedance response

**Fig. 1:**
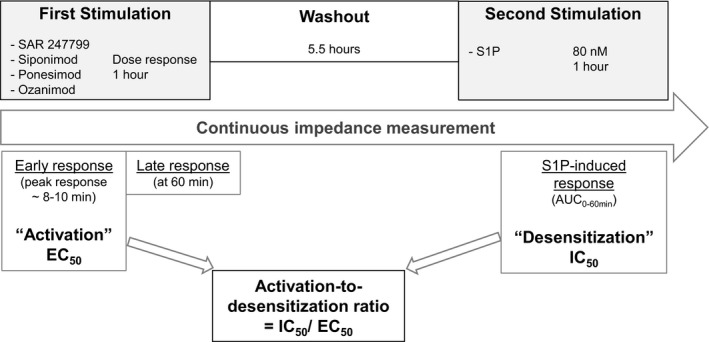
Protocol for impedance measurements.

**Fig. 2:**
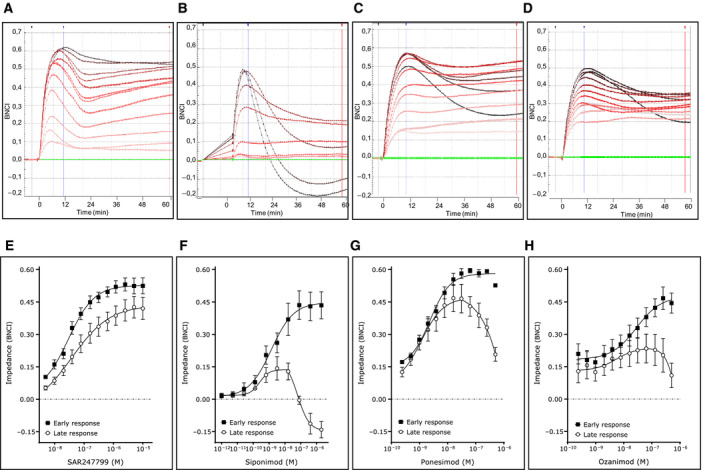
Early and late impedance responses of S1P_1_ agonists. Representative impedance profiles over 60 min for first stimulation with SAR247799 (A), siponimod (B), ponesimod (C), and ozanimod (D). Baseline responses (DMSO vehicle in absence of agonist) are denoted in green and increasing concentrations of the test agonists are denoted by increasing color intensity. Mean and SEM of separate experiments illustrating early and late responses of SAR247799 (E) *n* = 5, siponimod (F) *n* = 3, ponesimod (G) *n* = 3 and ozanimod (H) *n* = 3.

Electrical impedance was measured as an index of endothelial barrier integrity and expressed as baseline normalized cell index (BNCI). An overview of the experimental set‐up following stimulation of HUVECs with each test compound is illustrated in the schematic (Fig. 1). All compounds produced a rapid and concentration‐dependent increase in BNCI with a peak at approximately 8–10 min (Fig. 2A–D). After this peak response, the BNCI declined and the compounds showed differences in the kinetics of sustaining the BNCI response over the subsequent hour. This biphasic response was characterized by calculating the peak BNCI response, referred to as the early response, and the BNCI at 60 min, referred to as the late response.

For the early response, all compounds showed a concentration‐dependent response, with similar *E*_max_ between the 4 compounds (Fig. 2E–H). The potency of each compound in the early response was expressed as its EC_50_ and was between 1 and 30 nm for the 4 compounds (Table 1).

**Table 1:** Curve parameters for early‐phase, late‐phase, and desensitization responses.

Compound	*n*	Early phase				Late phase				Desensitization				Activation‐to‐desensitization ratio [95% CI]
		EC_50_ (nm) [95% CI]	Min	Max	Hill slope	EC_50_ (nm) [95% CI]	Min	Max	Hill slope	IC_50_ (nm) [95% CI]	Min	Max	Hill slope	
SAR247799	5	**26.1** [15.0–45.6]	0.101	0.527	1.0	**42.8** [25.0–73.3]	0.045	0.425	0.7	**2980** [1820–4880]	1.1	89.3	−1.9	**114** [91.1–143]
Ponesimod	3	**2.41** [1.03–5.61]	0.175	0.582	1.2	ND	–	0.468	–	**18.4** [1.01–336]	−38.2	79.3	−0.9	**7.66** [3.41–17.2]
Ozanimod	3	**26.8** [4.90–147]	0.188	0.461	1.1	ND	–	0.234	–	**170** [25.9–1120]	−13.9	91.5	−1.5	**6.35** [3.21–12.5]
Siponimod	3	**0.977** [0.200–4.76]	0.012	0.440	0.7	ND	–	0.150	–	**0.167** [0.00279–10.02]	−16.3	96.8	−1.0	**0.170** [0.0523–0.555]

*n*, separate experiments. EC_50_s, IC_50_s, and activation‐to‐desensitization ratio expressed as geometric means. Other parameters expressed as arithmetic means. Curve parameters not determined (ND) for late responses of siponimod, ponesimod, and ozanimod due to bell‐shaped responses (except for maximum response which was determined manually).

For the late response, SAR247799 displayed concentration‐dependent increases that paralleled the early response (Fig. 2E). The late response with SAR247799 gave an *E*_max_ that was 81% of that achieved in the early phase, and the EC_50_ values were similar (42.8 nm versus 26.1 nm) (Fig. 2E, Table 1).

The concentration–effect relationships in the late response for siponimod, ponesimod, and ozanimod were bell‐shaped, with maximum BNCI achieved at intermediate points in the concentration range (Fig. 2F–H). Siponimod, ponesimod, and ozanimod gave, respectively, maximum BNCI values in the late response which were 34%, 81%, and 51% of the *E*_max_ reached in the early response (Fig. 2F–H, Table 1). Higher concentrations of siponimod, ponesimod, and ozanimod displayed a concentration‐dependent decline in BNCI in the late response. The highest concentrations of siponimod caused endothelial barrier disruption as the BNCI values in the late response were below the baseline (Fig. 2F).

### β‐arrestin signaling contributes to the late response and barrier disruption

**Fig. 3:**
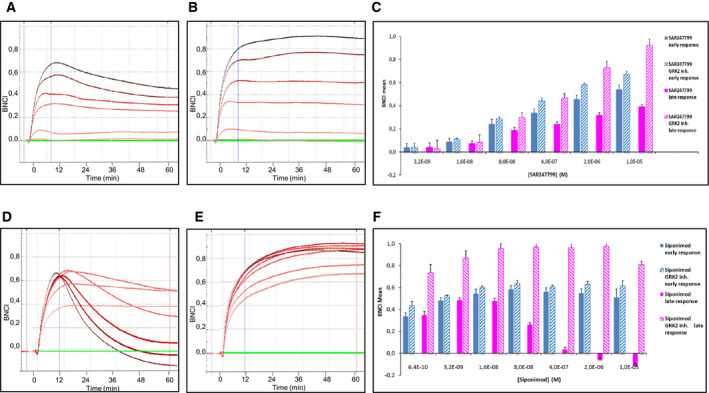
Contribution of β‐arrestin pathway activation to impedance profile. Representative impedance profiles over 60 min for first stimulation with SAR247799 and siponimod (A, D, respectively) and their influence by presence of GRK2 inhibitor (B, E). Baseline responses (DMSO vehicle in absence of agonist) are denoted in green, and increasing concentrations of the test agonists are denoted by increasing color intensity. Mean and SEM of 3 separate experiments for SAR247799 (C) and siponimod (F) displaying the early and late responses, in the presence or absence of GRK2 inhibitor.

The BNCI increase following S1P_1_ agonist stimulation has previously been shown to be Gi‐mediated [19]. Given that impedance responses provide an integrated assessment of ligand activity [20, 21] we sought to determine the contribution of β‐arrestin pathway activation to maintaining the Gi‐mediated BNCI increases. We did this by inhibiting the β‐arrestin pathway with a G protein‐coupled receptor kinase (GRK) inhibitor. GRKs cause intracellular phosphorylation of GPCRs, a requisite step for β‐arrestin binding and subsequent halting of G protein‐mediated activation [22]. GRK2‐mediated phosphorylation of S1P_1_ is also a requisite step for lymphopenia induced by S1P_1_‐desensitizing agents [23]. The GRK2 inhibitor had an IC_50_ of 44 nm in the GRK2 kinase assay, and it inhibited β‐arrestin recruitment in S1P_1_‐overexpressing cells with an IC_50_ of 1.1 µm. Thus, 10 µm of the GRK inhibitor was used for assessing the effect of inhibiting the β‐arrestin pathway on the impedance response of S1P_1_ agonists. For this evaluation, we chose SAR247799 and siponimod because they displayed the most‐sustained and the most‐transient BNCI increases, respectively. In the presence of the GRK2 inhibitor, the responses of siponimod and SAR247799 were no longer biphasic but showed a sustained BNCI increase with no signal decline over 60 min (Fig. 3A–E). The GRK inhibitor had little effect on the early response (at 10 min) of either compound (Fig. 3C,F). The late response of SAR247799 showed a concentration‐dependent increase in the absence of the GRK inhibitor, and this was increased a further 2‐fold by the presence of the GRK inhibitor (Fig. 3A–C). The late response of siponimod showed a concentration‐dependent decrease to negative BNCI values in the absence of the GRK inhibitor (Fig. 3D). We showed that these siponimod‐induced barrier‐disruptive properties were due to β‐arrestin activation, because in the presence of the GRK inhibitor the same concentrations of siponimod demonstrated improved barrier integrity (Fig. 3E,F).

### Concurrent activation and desensitization measurements reveal differences among compounds

**Fig. 4:**
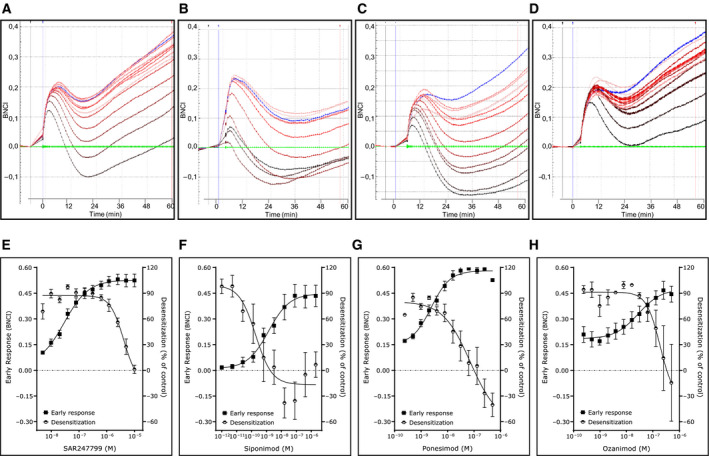
Effect of S1P_1_ agonists on desensitization of S1P‐induced impedance responses. Representative impedance profiles over 60 min for second stimulation with S1P (80 nm fixed concentration) following a first stimulation with increasing concentrations of SAR247799 (A), siponimod (B), ponesimod (C), and ozanimod (D). The baseline response (second response of vehicle following a first incubation with vehicle) is denoted in green. Control response (second response with S1P following a first incubation with vehicle) is shown in blue. S1P response following increasing concentrations of the test agonists in the first incubation are denoted by increasing color intensity. Mean and SEM of separate experiments illustrating the desensitization response (S1P‐induced response in the second stimulation) and the early response (first stimulation) of SAR247799 (E) *n* = 5, siponimod (F) *n* = 3, ponesimod (G) *n* = 3, and ozanimod (H) *n* = 3.

To measure the ability of each compound to desensitize S1P_1_, plates of the same compound‐treated cells used for measurement of early and late responses (first stimulation) were washed and then tested for their ability to mount a second BNCI response to a single concentration of the endogenous ligand S1P (second stimulation) (Fig. 1). As S1P, unlike most synthetic agonists, displays sustained impedance responses through S1P lyase‐dependent receptor recycling [19], the S1P‐induced impedance response was characterized by the area under the curve (AUC) over 60 min. At the highest concentrations tested, preincubation of all 4 compounds fully desensitized the S1P‐induced BNCI response (Fig. 4A–H). The highest concentrations of ponesimod caused, in the second stimulation stage, the S1P‐induced BNCI response to fall below the baseline (Fig. 4C,G), indicating that ponesimod not only blocked the S1P‐induced barrier‐promoting effect, but caused barrier disruption. To enable a comparison of the desensitization effect of compounds that had differing maximal effects, IC_50_s for the desensitization response were calculated as the concentration causing an absolute 50% reduction of the control S1P‐induced BNCI response (i.e., in the absence of test compound).

**Fig. 5:**
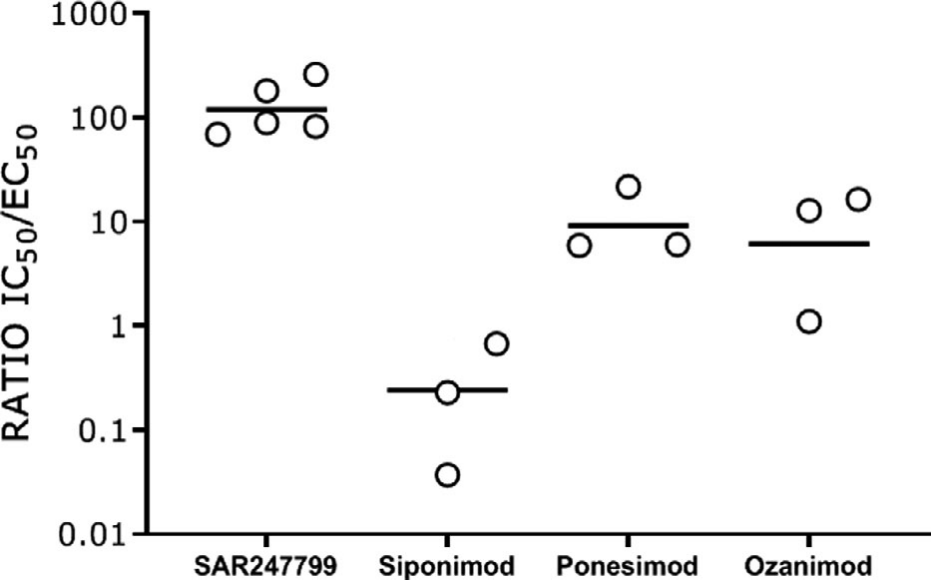
Activation‐to‐desensitization ratios of S1P_1_ agonists. Geometric means with 95% confidence intervals (SAR247799 *n* = 5, siponimod *n* = 3, ponesimod *n* = 3, and ozanimod *n* = 3).

The desensitization IC_50_s were compared to the activation EC_50_ in the early response and expressed as an activation‐to‐desensitization ratio (IC_50_/EC_50_). Siponimod was more potent in the desensitization assay (IC_50_ = 0.167 nm) than in the early‐phase activation assay (EC_50_ = 0.977 nm), giving an activation‐to‐desensitization ratio of 0.170 (Fig. 4F, Table 1, Fig. 5). Ponesimod, ozanimod, and SAR247799 were less potent in the desensitization assay compared to the early‐phase activation assay (Fig. 4G,H,, respectively), and the respective activation‐to‐desensitization ratios were 7.66, 6.35, and 114 (Table 1, Fig. 5).

## Discussion

This study describes a quantitative approach to characterize the activation‐to‐desensitization ratio for S1P_1_ modulators using an endothelial electrical impedance assay. The rank order of the 4 clinical compounds evaluated was SAR247799>ponesimod>ozanimod>siponimod with activation‐to‐desensitization ratios of 114, 7.66, 6.35, and 0.170, respectively. Consequently, SAR247799 had the best ability to activate S1P_1_, while minimizing S1P_1_ desensitization, and siponimod had the best ability to desensitize S1P_1_, while minimizing S1P_1_ activation.

A particular advantage of this method, due to the continuous nature of impedance monitoring, was that the same cell experiment was capable of measuring activation and desensitization properties; the cells were simply washed and re‐stimulated between the activation and desensitization parts of the study. It is well recognized that experimental parameters such as cell density, cell passage, cell viability as well as compound dilution can introduce variability to measurements in cell‐based assays. The experimental procedure controlled these variables by concurrent measurement of activation and desensitization using the same cells and compound dilution. As a result, bias ratios were reproducible between replicate experiments. Similar quantitative approaches could be utilized to compare other receptors endogenously expressed in HUVECs or, for that matter, other cell types.

The BNCI increase caused by S1P_1_ modulators in HUVECs is pertussin toxin‐sensitive and hence Gi‐mediated [19], consistent with BNCI increases seen with other Gi‐coupled receptor ligands [24, 25]. SAR247799 was able to sustain the impedance response over 60 min consistent with sustained Gi activation. However, the other compounds had markedly lower, and sometimes below baseline, impedance responses at 60 min, consistent with a study performed with ponesimod [19]. An inhibitor of β‐arrestin pathway signaling (GRK inhibitor) modified the BNCI profile to one of sustained activation over 60 min, confirming that β‐arrestin activation was responsible for reducing the late‐phase response. This finding is consistent with our previous characterization of SAR247799 relative to siponimod; SAR247799 activated G protein pathways more effectively than β‐arrestin or receptor internalization and SAR247799 was more G protein‐biased than siponimod [11]. Consequently, characterization of S1P_1_ agonists for their ability to sustain impedance responses could be a useful tool to rapidly distinguish between the biased nature of ligands. Quantitatively, there was a larger differential between siponimod and SAR247799 in the HUVEC activation‐to‐desensitization ratios compared to the bias ratios determined in S1P_1_‐overexpressing cells (cAMP versus β‐arrestin or cAMP versus internalization) [11], emphasizing some of the limitations of recombinant assays.

The BNCI response in some cases went below the baseline at the highest concentrations tested. This was particularly evident in the late‐phase response for siponimod and in the S1P_1_ desensitization setting for ponesimod. Positive BNCI values represent a tightening of the endothelial barrier, whereas negative BNCI represents barrier disruption. Disruption of the endothelial barrier has been reported with high doses of S1P_1_‐desensitizing molecules in cell‐based assays as well as in animals, particularly in the lung [11, 26, 27, 28]. It is also evidenced in clinical trials where dose‐dependent lung dysfunction and macular edema have been noted with various molecules [3, 4, 5, 29, 30]. These safety findings are particularly relevant in settings where lung endothelial barrier protection is actually desired (e.g., acute lung injury or systemic lupus erythematosus), and the disruption of barrier integrity assessed with BNCI values falling below baseline could be a potential approach to predict and mitigate this.

Lymphocyte reduction through S1P_1_ desensitization may not be the sole mechanism contributing to efficacy of S1P_1_ modulators in multiple sclerosis patients [31]. It has been proposed that an alternative mechanism to limit the entry of inflammatory cells into the CNS with this drug class is through improvement of blood–brain barrier (BBB) integrity through activation of S1P_1_ receptors on endothelial cells and astrocytes, which are the main cellular constituents of the BBB [32]. The recently approved dose of ozanimod in MS is associated with only 55% lymphocyte reduction, whereas fingolimod and siponimod produce 70–80% lymphocyte reduction at their approved doses. As we showed here that ozanimod is more biased than siponimod toward S1P_1_ activation, it is possible that a contribution of non‐S1P_1_‐desensitizing mechanisms, such as S1P_1_‐mediated endothelial/astrocyte protection, could explain why the efficacious doses were associated with different levels of lymphocyte reduction.

Endothelial protection as well as lymphocyte reduction could be desirable mechanisms to target in indications beyond MS. Chronic rheumatic disorders such as systemic lupus erythematosus, psoriasis, or systemic sclerosis are characterized by prominent endothelial dysfunction and marked vascular dysfunction, particularly in the microcirculation, suggesting that drug targeting the endothelium could find therapeutic utility in these conditions [33, 34]. However, lymphocytes clearly play an important role in these diseases as shown by the success of B cell depletion and IL17/IL23 pathway inhibition in systemic lupus erythematosus and psoriasis, respectively [35, 36]. Recently, a S1P_1_ modulator, cenerimod, demonstrated promising signals in a lupus trial at lymphocyte‐reducing doses [7], raising the question about relative contributions of lymphocyte‐reducing and endothelial‐protective mechanisms to the effects observed. Our approach to quantifying activation‐to‐desensitization ratios allows a new dimension to understanding, rationalizing, and potentially predicting relative differences in efficacy and suitability of various molecules targeting this pathway in the clinic.

In addition to the roles of S1P_1_ activation and S1P_1_ desensitization in endothelial barrier integrity and lymphocyte reduction, respectively, S1P_1_ activation also has heart rate‐reducing effects through its action on atrial myocytes. To achieve S1P_1_‐activating effects on the endothelium preferentially over the heart would require compounds with low tissue penetration or low volume of distribution. For this reason, the activation‐to‐desensitization ratios need to be considered in the context of tissue distribution properties. Table 2 summarizes both of these dimensions for the 4 compounds evaluated. SAR247799 has the highest S1P_1_ activation‐to‐desensitization ratio, as well as the lowest volume of distribution (7–23 L) compared to siponimod (124 L) [4], ponesimod (160 L) [37], and ozanimod (5590 L) [5]. It is noteworthy that ozanimod and ponesimod, although having similar activation‐to‐desensitization ratios, have a marked difference in their tissue distribution properties.

**Table 2:** Differences in G protein‐biased pharmacology and tissue distribution properties among clinical S1P_1_ agonists.

Compound	S1P_1_ activation‐to‐desensitization ratio in HUVEC	Volume of distribution in human (L) [Reference]
SAR247799	114	7–23 [13]
Ponesimod	7.66	160 [37]
Ozanimod	6.35	5590 [5]
Siponimod	0.170	124 [4]

In conclusion, this is the first study to compare and distinguish between the activation‐to‐desensitization properties of clinical S1P_1_ modulators. SAR247799 had the most suitable profile for endothelial protection, whereas siponimod had the best profile for S1P_1_ desensitization (and resulting lymphocyte reduction). As there are different therapeutic benefits associated with activating and desensitizing this receptor, the findings have clinical implications for selecting molecules in the class for desired effects on the endothelium versus on lymphocytes, respectively.

## Conflict of interest

At the time of conduct of the studies, all authors were employees of Sanofi. Authors affiliated with Sanofi may have equity interest in Sanofi. AAP and PJ are inventors of US patent number 9,782,411.

## Author contributions

PG and AAP conceived and designed the studies, PG and AB performed the experiments, PB and PJ acquired funding and supervised the work, PG, PB, AAP, and PJ analyzed and interpreted the results, PG and AAP wrote the manuscript, AB, PB, PJ reviewed and edited the manuscript, all authors were accountable for accuracy, integrity and approval of the final version.

## Data Availability

All relevant data supporting the conclusions are provided within the paper. Raw data can be obtained from the corresponding author upon reasonable request.
